# Chemical Biology, Molecular Mechanism and Clinical Perspective of γ-Secretase Modulators in Alzheimer’s Disease

**DOI:** 10.2174/157015911798376352

**Published:** 2011-12

**Authors:** Bruno Bulic, Julia Ness, Stefanie Hahn, Andreas Rennhack, Thorsten Jumpertz, Sascha Weggen

**Affiliations:** 1 Research Group Chemical Biology of Neurodegenerative Diseases, Center of Advanced European Studies and Research, D-53175 Bonn, Germany; 2 Molecular Neuropathology Group, Department of Neuropathology, Heinrich-Heine-University, D-40225 Duesseldorf, Germany

**Keywords:** Alzheimer's disease, neurodegeneration, amyloid-β peptide, gamma-secretase, gamma-secretase modulators.

## Abstract

Comprehensive evidence supports that oligomerization and accumulation of amyloidogenic Aβ42 peptides in brain is crucial in the pathogenesis of both familial and sporadic forms of Alzheimer's disease. Imaging studies indicate that the buildup of Aβ begins many years before the onset of clinical symptoms, and that subsequent neurodegeneration and cognitive decline may proceed independently of Aβ. This implies the necessity for early intervention in cognitively normal individuals with therapeutic strategies that prioritize safety. The aspartyl protease γ-secretase catalyses the last step in the cellular generation of Aβ42 peptides, and is a principal target for anti-amyloidogenic intervention strategies. Due to the essential role of γ-secretase in the NOTCH signaling pathway, overt mechanism-based toxicity has been observed with the first generation of γ-secretase inhibitors, and safety of this approach has been questioned. However, two new classes of small molecules, γ-secretase modulators (GSMs) and NOTCH-sparing γ-secretase inhibitors, have revitalized γ-secretase as a drug target in AD. GSMs are small molecules that cause a product shift from Aβ42 towards shorter and less toxic Ab peptides. Importantly, GSMs spare other physiologically important substrates of the γ-secretase complex like NOTCH. Recently, GSMs with nanomolar potency and favorable *in vivo* properties have been described. In this review, we summarize the knowledge about the unusual proteolytic activity of γ-secretase, and the chemical biology, molecular mechanisms and clinical perspective of compounds that target the γ-secretase complex, with a particular focus on GSMs.

## INTRODUCTION

### The Amyloid Hypothesis of Alzheimer's Disease Provides a Rationale for the Development of Disease-Modifying Therapies

Alzheimer’s disease (AD) is the most common age-related neurodegenerative disorder currently affecting 20-30 million individuals worldwide. With increasing life expectancy, the European region alone will harbor around 11.2 million prevalent AD cases by 2050 [1]. The cardinal symptom of the disease is progressive memory loss due to the degeneration of neurons and synapses in the cerebral cortex and subcortical regions of the brain. Neuropathologically, AD is characterized by the extracellular deposition of amyloid-β (Aβ) peptides, neurofibrillary tangle formation, a chronic inflammatory response and oxidative damage [2].

**Fig. (1):**
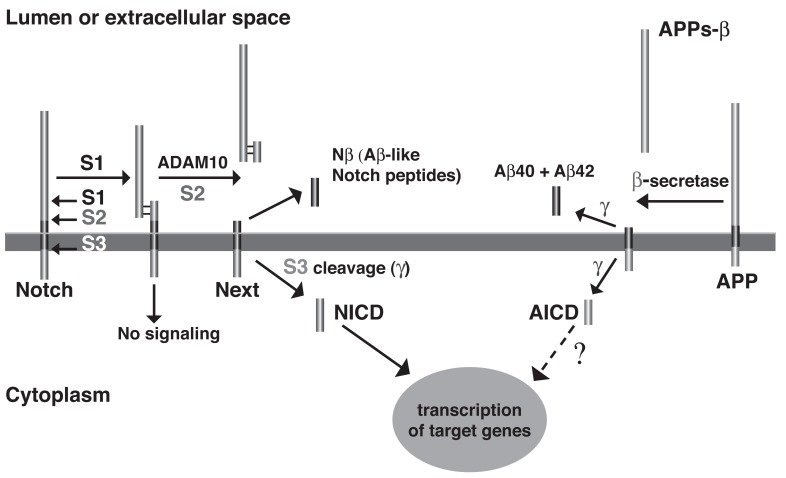
Proteolytic processing of APP and the NOTCH receptor by γ-secretase. The Aβ peptide is derived by sequential proteolysis from APP, a ubiquitously expressed type I transmembrane protein. In the amyloidogenic pathway, APP molecules are first cleaved at the cell surface or in early endosomes by β-secretase (BACE1), a membrane bound aspartyl protease, generating a large, soluble ectodomain, APPs-β, and a membrane-bound fragment, C99, that defines the N-terminus of the Aβ sequence [197]. Subsequent cleavage of C99 by the aspartyl protease γ-secretase approximately in the middle of the TMD generates the C-terminus of the Aβ peptide and releases Aβ from APP. γ- secretase generates Aβ peptides of varying length elongated or truncated at the C-terminus, with peptides ending after 40 and 42 amino acids being the predominant species. In addition to cleavage in the middle of the TMD (γ-cleavage), γ-secretase cleaves close to the cytosolic border of the membrane (ε-cleavage). This cleavage liberates the APP intracellular domain (AICD), which may have a function in transcriptional regulation [42]. The NOTCH receptor is synthesized as a 300 kDa precursor that is cleaved by a furin-like convertase in the *trans*- Golgi compartment and assembled into a mature heterodimer receptor through non-covalent linkage of the resulting protein fragments (S1 cleavage) [27, 28]. At the cell surface, binding of DSL family ligands (Delta/Jagged) induces shedding of the ectodomain by the metalloprotease ADAM10 (S2 cleavage). The resulting, membrane-bound C-terminal fragment termed NOTCH extracellular truncation (NEXT) undergoes cleavage by γ-secretase at the cytosolic border of the membrane (S3 cleavage), which releases the NOTCH intracellular domain (NICD) into the cytosol. NICD travels to the nucleus where it functions as a transcriptional activator [27, 28]. Interestingly, γ-secretase also cleaves the NEXT fragment in the middle of the TMD at sites topologically similar to the cleavage sites in APP that generate the Aβ peptides. These cleavages (S4 cleavage) generate the Nβ peptides, predominantly Nβ21 and Nβ25, which are released into the extracellular space [113]. In addition to NOTCH receptors, a large number of type-1 membrane proteins including NOTCH receptor ligands, APP homologs, ErbB-4, E- and N-cadherin and CD44 have been identified as γ-secretase substrates. Whereas the physiological relevance of γ-secretase-mediated cleavage events in many of these substrates remains to be clarified, suppression of NOTCH receptor processing and signaling has been shown to result in dramatic phenotypes in a variety of organisms [26].

Comprehensive evidence supports the amyloid hypothesis of AD, which states that aberrant production, aggregation and deposition of Aβ plays a causal role in the pathogenesis [2-4]. Aβ is a proteolytic fragment, which is generated through sequential cleavage of the amyloid precursor protein (APP) by β- and γ-secretase (Fig. **1**). Cells produce Aβ peptides of variable length, but peptides of 40 and 42 amino acids are the most prevalent species. Genetic, toxicology and animal studies strongly indicate that the longer, highly amyloidogenic Aβ42 isoform is the key pathogenic species and could be an ideal therapeutic target in AD [2-4]. Most importantly, genetic analysis of familial forms of AD with early disease onset (FAD) has demonstrated that modest overproduction of amyloidogenic Aβ42 peptides in the brain is sufficient to cause this devastating neurodegenerative disease with complete penetrance [5]. Interestingly, it appears that a high ratio of Aβ42 to Aβ40 peptides is critical for the formation of neurotoxic Aβ oligomers whereas the total amount of Aβ42 peptides produced is a less important determinant. In addition, it has been demonstrated that Aβ40 can antagonize the aggregation rate and neurotoxic properties of Aβ42 both *in vitro* and in animal models of AD [6-10]. Finally, in line with the observation that the common, age-associated forms of AD are clinically and histopathologically remarkably similar, it has been argued that oligomerization and accumulation of toxic Aβ42 peptides in brain is also the triggering event in the sporadic forms of the disease [2, 4, 11].

Neuropathology studies indicate that brain accumulation of Aβ likely starts 10-20 years before the clinical onset of AD [12]. Furthermore, amyloid imaging studies with positron emission tomography suggest that Aβ deposition begins insidiously in cognitively normal individuals, and that subsequent cognitive decline and neurodegeneration may proceed independently of Aβ accumulation [13]. Today, neuropsychological testing provides around 85% accuracy in the diagnosis of AD. However, this diagnosis is based on substantial cognitive impairments that severely interfere with activities of daily life, and it is known that patients at this stage have already suffered extensive neuronal damage [14]. Taken together, these findings indicate that the future of AD therapeutics is not treatment in advanced stages but primary and secondary prevention of the disease. However, for disease prevention to succeed, any potential intervention should target the key mechanisms that are responsible for initiation of the disease process. In addition, a drug can have only minimal side effects and needs to be tolerated for long periods of time, potentially decades in the case of AD. Despite considerable progress in the understanding of AD pathology, only symptomatic treatments are available today [15]. The most advanced efforts to develop disease-modifying therapies for AD have focused on suppressing Aβ production with small molecule inhibitors of β- and γ-secretase, on enhancing Aβ clearance by immunization protocols, and on the development of compounds that prevent aggregation of Aβ peptides. However, none of these approaches have so far yielded clinically viable therapies, and all of them are confronted with technical challenges and safety concerns [15].

In conclusion, the amyloid hypothesis has undergone revisions in recent years, but remains the only concept that can conclusively explain the disease course in both familial and sporadic forms of AD [4, 16]. The realization that Aβ accumulation might trigger the disease process many years before the development of clinical symptoms demands early intervention in cognitively normal individuals. This has created novel challenges to develop intervention strategies that prioritize safety. However, this also holds the promise that effective anti-amyloidogenic interventions through safe pharmaceuticals might be able to halt the disease process before any cognitive dysfunction occurs.

### Overview of Compound Classes Targeting the γ-Secretase Complex

The proteases that process APP and generate the Aβ peptides remain the most straightforward targets for anti-amyloidogenic intervention strategies. β-Secretase is an attractive target that may have a favorable side effect profile, but inhibitor development has proven exceedingly difficult, and only a single compound has reached clinical trials so far [17]. In contrast, drug development for the aspartyl-protease γ-secretase has progressed rapidly. γ-Secretase has a complicated multi-subunit architecture with overall 19 transmembrane domains (TMDs), and no high-resolution structural data for this protease is available, which indicates that drug design has relied more on high-throughput screening and serendipity than on rational drug design. Nevertheless, a large number of small molecule γ-secretase inhibitors (GSIs), some with subnanomolar affinity and excellent *in vivo* properties, have been developed [17]. According to their binding sites within the γ-secretase complex, these GSIs have been categorized into three major classes; active site, docking site and allosteric site binders (Table **1**). The first generation of active site directed GSIs such as L-685,458 (Fig. **4**) were derivatives of HIV protease inhibitors with a characteristic unhydrolysable hydroxyethylene mimicking the substrate amide bond [18]. Interestingly, these peptide-based compounds show little similarity with the residues within the APP-cleavage site, and attempts to improve the inhibitory potency using APP-mimics were initially unsuccessful [19]. However, subsequent introduction of α-helix-inducing residues in the sequence provided highly potent inhibitors, which were found to bind to an exosite also called substrate docking site in the enzyme complex (Fig. **5**) [20]. Efforts to circumvent the poor pharmacokinetics generally observed with peptides led to a second generation of GSIs, developed on the DAPT scaffold (*N*-(*N*-(3,5-difluorophen-acetyl)-L-alanyl)-*S*-phenylglycine t-butyl ester, (Fig. **6**). These allosteric site binders present a difluorobenzyl-dipeptide backbone, with numerous modifications on the C-terminal substituents [21-25]. Further development has also yielded aryl-sulfonamide GSIs such as MRK-560 (Fig. **7**), which likely target the same allosteric binding site [21-24].

Unfortunately, early animal studies with GSIs from these classes have uncovered severe mechanism-based toxicity [17, 26]. Besides APP, more than 50 other substrates of γ-secretase have been identified, but it appears that the toxicity caused by GSIs can be largely explained by the essential role of γ-secretase in proteolytic processing of one specific substrate, the NOTCH receptor. Similarly to APP, the NOTCH receptors undergo intramembrane cleavage by γ-secretase, releasing the NOTCH intracellular domain (NICD) to regulate transcription of target genes involved in cell fate decisions during embryogenesis but also in mitotic cell populations of adult mammals including lymphocytes and intestinal epithelial cells (Fig. **1**) [27, 28]. The majority of GSIs indiscriminately block cleavages within the TMDs of γ-secretase substrates and prevent Aβ and NICD formation with equal potency. Inevitably, γ-secretase treatment in mice at doses that effectively reduced Aβ levels in brain caused severe hematopoietic phenotypes and gastrointestinal toxicity [17, 26]. Nevertheless, one non-selective GSI, the DAPT-like LY-450,139 (Semagacestat, Fig. **6**) has been explored in a Phase III clinical trial in AD patients. However, clinical development of this compound was recently terminated due to enhanced cognitive decline and an increased risk of skin cancer in the treatment group (Eli Lilly and Company, press release August 17, 2010).

Importantly, two newer classes of small molecules, *NOTCH-sparing GSIs* and γ-secretase modulators (GSMs), have revitalized γ-secretase as a drug target in AD. NOTCH-sparing GSIs such as BMS-708,163 and GSI-953 (Begacestat) are structurally highly similar to non-selective sulfonamide GSIs (Fig. **7**). These compounds block all γ-secretase-mediated cleavage events in the APP TMD but have been reported to avoid effects on NOTCH processing in a certain range of concentrations [24, 29]. A distinct class of NOTCH-sparing compounds has structural similarities to kinase inhibitors (Fig. **8**). Very little is known about the mode of action of these compounds, but they have been shown to interact with a putative nucleotide-binding domain within the γ-secretase complex [30-32]. Finally, GSMs are small molecules that cause a product shift from the highly amyloidogenic Aβ42 peptide towards shorter and less toxic Aβ peptides [26]. These fascinating molecules also spare processing and signaling of the NOTCH receptor. Importantly, the fact that GSMs change the cleavage pattern but leave the overall processing activity of the protease unaffected indicates that GSMs may generally maintain the function of physiologically important γ-secretase substrates. The first GSMs were discovered in the class of non-steroidal anti-inflammatory drugs (NSAIDs) (Fig. **9**) [33]. However, NSAID GSMs such as sulindac sulfide or ibuprofen are plagued by pharmacological liabilities such as low potency and brain permeability, as well as by side effects related to cyclooxygenase inhibition [34]. More recently, GSMs with nanomolar potency and favorable *in vivo* properties have been described [35]. These compounds belong to two major classes, acidic GSMs with structural similarities to NSAID (Figs. **12**, **13**, **16**) and non-acidic bridged aromatics like E-2012 (Fig. **14**). The molecular details how GSMs modulate the activity of γ-secretase remain largely undefined, with conflicting data supporting a binding site within the substrate APP or within subunits of the γ-secretase complex [36-38].

In summary, intense research in both academia and pharmaceutical companies has helped to alleviate early safety issues and has assured that γ-secretase remains one of the most promising therapeutic targets in AD. In the following, we summarize the knowledge about the unusual proteolytic activity of γ-secretase, and review the chemical biology and clinical perspective of compounds that target the γ-secretase complex with a particular emphasis on GSMs.

## MOLECULAR MECHANISM OF γ-SECRETASE INHIBITORS AND MODULATORS

### γ-Secretase and the Molecular Mechanism of Aβ Generation

γ-Secretase belongs to the intramembrane-cleaving proteases (I-CLiPs) that hydrolyze peptide bonds of their substrates in the membrane, and comprise zinc metalloproteases, serine proteases and aspartyl-proteases [39]. γ-Secretase is a multi-subunit aspartyl I-CLiP with the presenilin (PSEN) proteins, either PSEN1 or PSEN2, at its catalytic core [40-42]. Current evidence indicates that PSEN has a nine transmembrane domain (TMD) topology. PSEN proteins are endoproteolytically cleaved during assembly and maturation of the γ-secretase complex into N- and C-terminal fragments that remain non-covalently associated. The PSEN fragments are incorporated together with three accessory proteins, nicastrin, anterior pharynx defective-1 (APH-1) and presenilin enhancer-2 (PEN-2), into high molecular weight complexes that correlate with the bulk of enzymatic activity [40, 41]. The function of these accessory proteins is largely unknown. Controversial data supporting a role of nicastrin in substrate recognition has been published [43, 44], and more recently, it has been questioned whether nicastrin is at all essential for enzymatic activity of the γ-secretase complex [45, 46]. APH-1 and PEN-2 are highly hydrophobic proteins with altogether 9 TMDs and may serve structural functions in the complex. PEN-2 supports the stability of the PSEN fragments in the γ-secretase complex, and APH-1 has also been implicated in substrate interaction [47-49]. γ-Secretase most likely forms a monomeric complex with the four subunits present in a 1:1:1:1 stoichiometry [50]. The subunits assemble in a stepwise process in early compartments of the secretory pathway. Subsequently, the fully assembled γ-secretase complex travels to compartments of the late secretory pathway and the plasma membrane where it is functionally active [40-42].

**Fig. (2):**
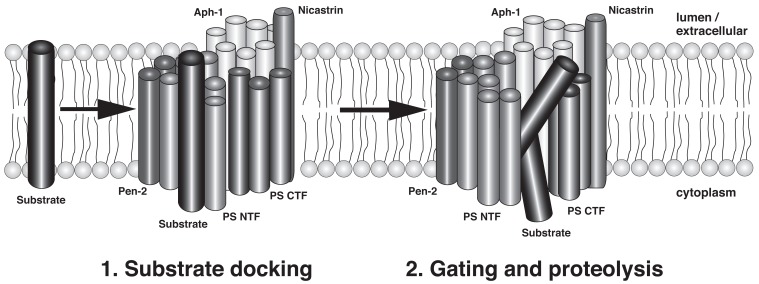
Hypothetical model of substrate interaction and processing by γ-secretase (modified after [74]; see text for details).

Two critical aspartate residues in TMD6 and TMD7 of PSEN form the active center of γ-secretase and are essential for enzyme activity [51]. However, at present we only have a partial understanding of how γ-secretase accomplishes the hydrolysis of peptide bonds in the hydrophobic environment of the membrane. In the absence of high-resolution structural data of the γ-secretase complex, cysteine scanning mutagenesis of PSEN1 has been employed to gain insight into the topology and catalytic mechanism of γ-secretase [52]. This technique involves the substitution of specific amino acid residues with cysteine and the subsequent labeling with sulfhydryl-directed reagents to assess the water accessibility of the cysteine residues. Cysteine residues located on the extracellular or cytosolic side of the membrane are readily water accessible and can be labeled by either membrane-permeable or impermeable labeling reagents. On the contrary, cysteines embedded in the membrane are not labeled by sulfhydryl-directed reagents unless they are exposed to a water-containing cavity. This type of analysis has demonstrated that TMDs 6 and 7 around the catalytic aspartate residues form a hydrophilic cavity within the membrane that may allow access for water molecules, which are required to hydrolyze peptide bonds [53, 54]. In addition, parts of TMD9 in the C-terminal half of PSEN are located in proximity to the catalytic site and may further contribute to substrate binding and delivery to the active site [55, 56]. It has been proposed that a lateral gating mechanism controls access of the membrane-anchored substrate to the hydrophilic catalytic cavity. This assumes that the substrate initially binds to a distinct substrate-binding site (docking site) on the outer surface of the γ-secretase complex and is subsequently transported into the active site and cleaved (Fig. **2**). Support for both a water accessible catalytic pore and a lateral gating mechanism in γ-secretase has also been derived from the available crystal structures of prokaryotic serine and metalloprotease I-CLiPs [39, 57-59]. How γ-secretase selects between different substrates is largely unknown, but mutation of a leucine two residues N-terminal from the catalytic aspartate in TMD7 has been demonstrated to block NOTCH processing and NICD generation without effect on Aβ and AICD generation [60], indicating that this residue is critically involved in the selection between the substrates NOTCH and APP.

**Fig. (3):**
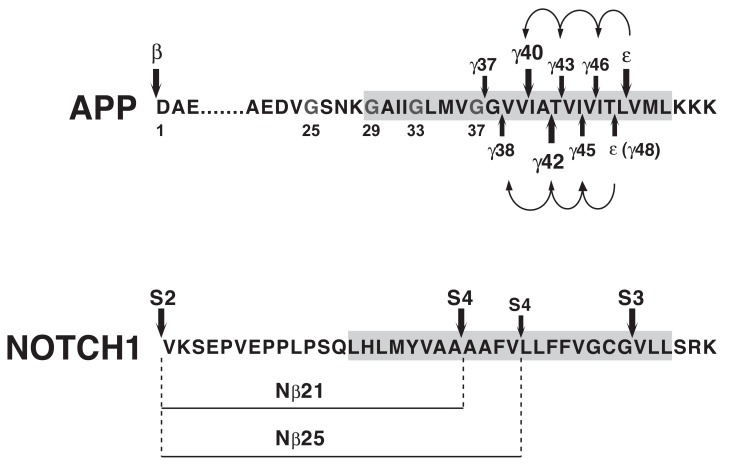
γ-secretase cleavage sites within the TMDs of APP and the NOTCH1 receptor. Cleavage by β-secretase generates the N-terminus of the Aβ sequence. Subsequently, γ-secretase cleaves within the APP TMD at multiple sites. According to the sequential cleavage model, cleavage occurs initially close to the cytosolic border of the TMD at the ε-site or at the alternative ε-site (γ48), and then in two product lines along opposite surfaces of the helical axis of the substrate APP. Most recently, direct evidence for this model has been provided with the detection of corresponding tripeptides that are released after each sequential cleavage step [68]. This study also described detection of a tetrapeptide that would explain conversion of Aβ42 to Aβ38. Evidence for conversion of Aβ40 to Aβ37 is lacking. Cleavage of NOTCH1 by ADAM10 (S2 cleavage) generates a membrane-bound, C-terminal fragment that is a direct substrate for γ-secretase. Cleavage occurs close to the cytosolic border of the TMD and results in release of the NICD domain (S3 cleavage). In addition, γ-secretase-mediated cleavage events in the middle of the TMD generate two peptides, Nβ21 and Nβ25, with differing C-termini analogous to the Aβ peptides (S4 cleavage) [113]. A similar dual-cleavage mechanism has been proposed for other substrates of γ-secretase [114, 198]. GSMs reduce the generation of Aβ42 and of the corresponding Nβ25 peptides, but spare γ-secretase cleavage at the ε-site and the S3-site and liberation of the AICD and NICD domains. This indicates that the topology of the γ-secretase cleavage sites within the TMD is crucial for the modulation of a given γ- secretase substrate by GSMs.

How exactly the Aβ peptides are generated by γ-secretase has not been fully resolved but recent progress supports a model of sequential cleavage events [61]. After cleavage of APP by β-secretase, which creates the N-terminus of the Aβ peptide sequence, γ-secretase cleaves at multiple sites within the APP TMD (Fig. **3**). Based on the abundance of individual cleavage products, predominant cleavage events occur after Val-40 (generating Aβ40) and after Ala-42 (generating Aβ42) approximately in the middle of the TMD, and after Leu-49 close to the cytosolic border of the TMD, which generates the AICD fragment (ε-cleavage) [40, 61]. Minor cleavages take place after various other residues, and less abundant Aβ peptides have been detected either in cell supernatants (Aβ37, Aβ38, Aβ39) or cell lysates (Aβ43, Aβ45, Aβ46, Aβ48) [62]. The latter peptides are highly hydrophobic and only inefficiently secreted. In addition, cleavage after Thr-48 produces a less abundant but longer AICD fragment (alternative ε-cleavage) [63]. The APP TMD has been proposed to adopt an α-helical conformation with 3.6 residues for one complete turn, and to interact on one surface with the active center of γ-secretase [64]. In this conformation cleavage sites for Aβ40, Aβ43, Aβ46 and Aβ49 (ε-cleavage) would align on one surface of the helix, and cleavage sites for Aβ38, Aβ42, Aβ45 and Aβ48 (alternative ε-cleavage) on the opposite surface (Fig. **3**) [62]. According to the sequential cleavage model as proposed by Ihara and colleagues, ε-cleavage would occur first with subsequent cleavages taking place sequentially at every 3 residues along the α-helical surface [62]. Two separate product lines of Aβ peptides would then depend on whether the first cleavage event occurred at the ε-cleavage site (Aβ49, Aβ46, Aβ43, Aβ40) or at the alternative ε-cleavage site (Aβ48, Aβ45, Aβ42, Aβ38) [62, 63, 65, 66]. While interdependency between ε-cleavage and the γ-secretase cleavage events in the middle of the APP TMD (generating Aβ40 and Aβ42) has been well documented [67], coordinated cleavages for other Aβ species, in particular Aβ48, Aβ45, Aβ42 and Aβ38, have only been insufficiently supported. However, most recently, direct evidence for the sequential cleavage model has been provided with the detection of corresponding tripeptides that are released after each sequential cleavage step by tandem mass spectrometry [68]. This study using a CHAPSO-reconstituted γ-secretase *in vitro* assay also described detection of a tetrapeptide that would explain conversion of Aβ42 to Aβ38, and is consistent with the idea that Aβ42 is a direct precursor of Aβ38 (Fig. **3**). Clearly though, the reconstituted γ-secretase *in vitro* assay displayed some features that are at odds with results from cellular assays. For example, only Aβ peptides up to Aβ40 and Aβ38 were produced in this assay. In contrast, tissue culture supernatants have been shown to contain shorter peptides such as Aβ37, Aβ34 and Aβ33, whose generation was suppressed by GSIs [69]. In addition, using cells expressing FAD PSEN1 mutants, evidence has been provided that Aβ42 and Aβ38 can be generated independently by γ-secretase arguing against a strict precursor product relationship between these peptides [35, 70]. Evidently, additional studies are required to reconcile results from γ-secretase *in vitro* assays and observations in cellular assays. Furthermore, it remains completely unresolved how the substrate relocates in the active center of γ-secretase to expose the following peptide bond during the sequential cleavage process. Evidence has also been provided for sequential cleavage of NOTCH by γ-secretase. In this γ-secretase substrate, cleavage at two major sites in the middle of the TMD (S4 cleavage) seems to depend on prior cleavage at the S3 site close to the cytosolic border of the TMD [71, 72]. These cleavage sites in NOTCH are topologically similar to the γ-secretase cleavage sites in APP, with the S4 cleavage sites resembling the Aβ40/Aβ42 cleavage sites and the S3 cleavage site being analogous to the ε-cleavage site in APP (Fig. **3**).

Another important issue is whether APP is processed by γ-secretase as a monomeric or a dimeric substrate. Around 25% of all γ-secretase substrates contain GXXXG motifs in their TMDs, which have been demonstrated to mediate TMD helix-helix association in some membrane proteins [73, 74]. However, in this respect, APP is unique as it contains three consecutive GXXXG motifs in its TMD and juxtamembrane domain (amino acids 25-37 of the Aβ sequence (Fig. **3**) [74]. Importantly, G/A or G/I mutations in the G_29_XXXG_33_ motif were shown to lower Aβ42 production and to increase shorter Aβ species, mimicking the effects of GSMs [75]. These glycine mutants further displayed reduced helix-helix interaction of the APP TMD in a bacterial dimerization assay. Based on these findings, it was proposed that sequential cleavage of APP by γ-secretase might be regulated by the dimerization strength of the APP TMD [75]. In a wild type APP substrate, γ-secretase cleavage would cleave from the ε-cleavage site in N-terminal direction until the dimerization motif would restrict the enzyme from proceeding further, causing termination of proteolysis and the release of the most prevalent Aβ40 and Aβ42 peptides. Attenuation of the dimerization strength by mutation of the glycine residues would allow γ-secretase to cleave further along the TMD, leading to the generation of shorter Aβ species and reduced Aβ42 production [75]. By inference, attenuation of APP dimerization could also be a plausible mechanism how GSMs shift Aβ42 production to shorter Aβ species (see below) [38]. However, whether APP dimerization generally facilitates Aβ production remains controversial [76-78]. Furthermore, evidence indicates that γ-secretase mediated proteolysis of other substrates might not be controlled by oligomerization [79].

One prediction of the sequential cleavage model is that, in principle, it should be possible to inhibit the γ-secretase cleavage events in the middle of the TMD that generate the Aβ peptides without blocking the ε-cleavage site or topologically similar cleavage sites in other γ-secretase substrates that generate the ICD domains. Indeed, evidence for this prediction have been provided that goes beyond the discovery of GSMs. Specific mutations in the APP luminal juxtamembrane domain were shown to dramatically lower Aβ production without effects on ε-cleavage in cell-based assays [80]. Furthermore, RNAi-mediated down-regulation of the γ-secretase associated protein TMP21 increased Aβ production but did not affect ε-cleavage in APP [81]. Hence, TMP21 might normally repress Aβ production without impairment of ICD formation from APP, NOTCH and other γ-secretase substrates. Mechanistically, these effects are different from the mode of action of GSMs as neither APP juxtamembrane mutations nor TMP21 downregulation caused any selective effects on Aβ42 production but instead affected overall Aβ production [80, 81]. In addition, this type of modulation is also different from the mode of action of NOTCH-sparing GSIs [29]. While no detailed data concerning their effects on APP and NOTCH processing have been published yet, current evidence suggests that these compounds do not discriminate between the APP cleavage sites and impede both Aβ and AICD production [24, 29, 82] similar to prototypical non-selective GSIs like DAPT (Table **1** and Fig. **6**). Instead, NOTCH-sparing GSIs seem to act preferentially on APP versus NOTCH *via *an unknown mechanism.

### γ-Secretase Inhibitors Interact with Presenilin, the Catalytic Subunit in the γ-Secretase Complex

Either through rational design or chemical library screens, large numbers of highly potent GSIs have been identified [17, 83]. Depending on their chemical structure and mechanism of action, these compounds seem to target three different binding sites in the γ-secretase complex that are all located within the PSEN proteins [20-24, 84-86]. The location and spatial relationship between these binding sites have been probed by several experimental approaches including photo-affinity labeling experiments, cross-competition studies and radioligand binding assays. 

**Fig. (4):**
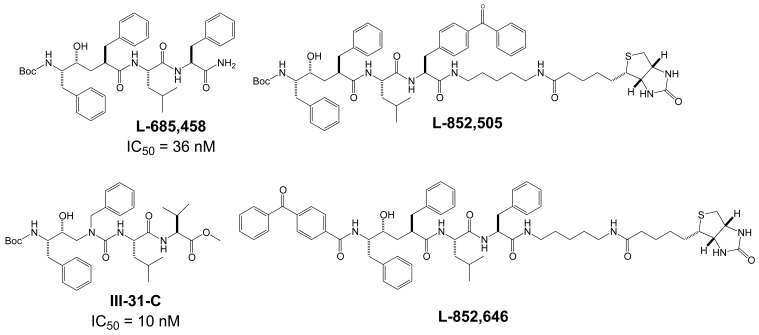
Structures of the active-site directed γ-secretase inhibitor L-685,458 and close analogues.

Photo-affinity labeling studies with two analogues of L-685,458 (Fig. **4**) based on the same unhydrolizable hydroxyethylene dipeptide isoster (L-852,505 and L-852, 646, Fig. **4**) and equipped with a photo-reactive benzophenone group (Fig. **4**) have demonstrated that active-site directed transition-state GSIs like L-685,458 interacted with both the N- and C-terminal fragments of PSEN1. While both L-858,505 and L-852,646 target the catalytic active site, L-852,505 labeled preferentially PSEN1-CTF whereas L-852,646 associated distinctly with PSEN1-NTF [85]. This finding is consistent with the idea that the active site of γ-secretase is located at the interface of the two PSEN fragments and is composed of one aspartyl residue in each subunit [84, 85]. 

**Fig. (5):**
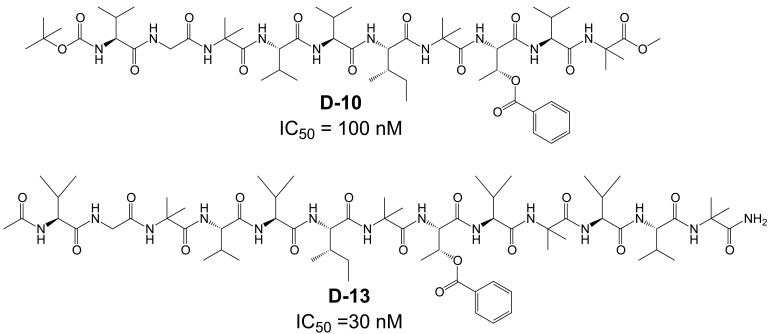
Structure of docking-site binders: α-helical peptide D-10 and the three-amino acids extended analogue D-13.

Furthermore, these transition-state inhibitors bind to the γ-secretase complex in a non-competitive fashion with respect to the substrate APP, and blocking the active site with a transition-state GSI did not prevent binding of substrate to the enzyme complex. Indeed, the substrate was found to co-purify with an enzyme-bound transition-state inhibitor [87, 88]. These results have led to the proposition that γ-secretase contains a docking site for substrate binding that is responsible for initial recognition of the substrate prior to movement to the catalytic site. Consequently, α-helical peptides based on the TMD of APP interacted with this docking site and were also potent inhibitors of γ-secretase [20]. Photo-labeling studies with α-helical peptides of variable length as well as mutagenesis studies have further indicated that the active site and the docking site are spatially close together and may even overlap [20, 60]. In this respect, it has been reported that a photo-reactive derivative of a 10-residues α-helical peptide D-10 (Fig. **5**) binds at the NTF-CTF interface, but could not be displaced by the transition state inhibitor III-31-C (Fig. **4**). However, displacement occurred with a three amino acids elongated D-13 analogue (Fig. **5**), suggesting close proximity of the active and docking sites [20]. 

**Fig. (6):**
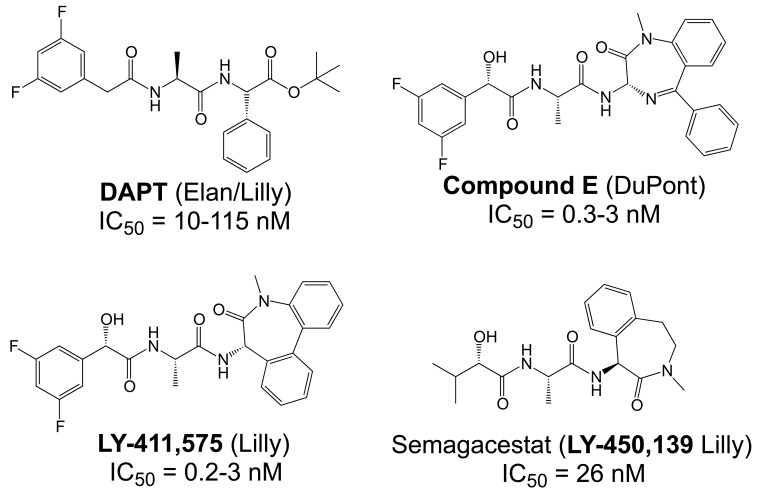
Structures of the allosteric site binder DAPT and derivatives.

**Fig. (7):**
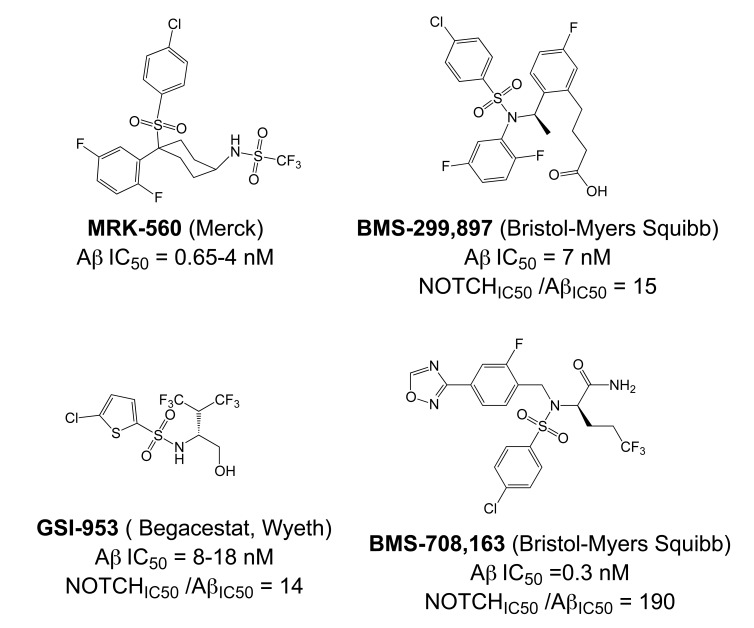
Structures of aryl sulfonamide-based γ-secretase inhibitors. Some of these compounds display selectivity for APP over NOTCH and have been termed NOTCH-sparing γ-secretase inhibitors.

Besides inhibitors targeting the active or docking sites described above, a third class of inhibitors has been identified. These inhibitors target a discrete binding site, and include the dipeptidic compounds DAPT (*N*-(*N*-(3,5-Difluorophenacetyl)-L-Alanyl)-*S*-Phenylglycine t-butylester, (Fig. **6**) and its C-terminally modified analogues extended with diazepines such as compound-E, or with caprolactams such as LY-411,575 and LY-450,139 (Semagacestat, Fig. **6**). Moreover, aryl sulfonamide GSIs such as MRK-560 and the third generation NOTCH-sparing GSIs BMS-299,897 and GSI-953 (Fig. **7**) may also target the same allosteric binding site. Specifically, a photo-activatable derivative of DAPT was shown to interact with a binding site in the PSEN1 CTF [21]. However, a follow up study investigated photo-activatable derivatives of two close analogs of DAPT and reported that these compounds target the PSEN1 NTF [86]. Together, these results indicate that the binding site for dipeptidic GSIs may also be located at the interface of the two PSEN fragments, and that labeling of either fragment may depend on the chemical space that a specific photo-probe occupies.

**Fig. (8):**
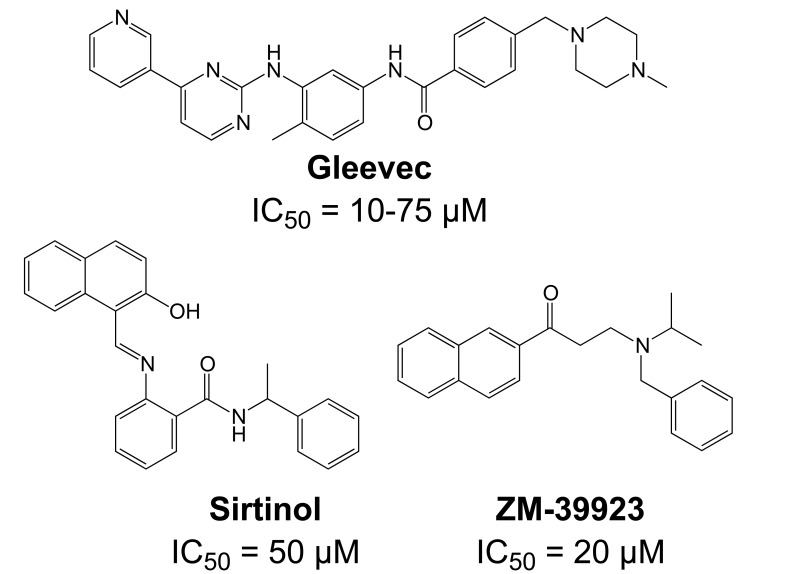
Structures of NOTCH-sparing γ-secretase inhibitors targeting a nucleotide-binding domain.

Although the synthesis of photo-probes based on active aryl sulfonamide GSIs has been described (Fig. **7**), no analogous binding studies have been reported yet for this class of compounds [89]. Nevertheless, several inhibitor cross-competition studies strongly suggest that all non-transition-state GSIs including the aryl sulfonamide NOTCH-sparing GSIs such as GSI-953 (Begacestat, Fig. **7**), 14-fold selectivity for APP over NOTCH), BMS-299,897 (Fig. **7**), 15-fold selectivity APP over NOTCH) [90] and BMS-708,163 (Fig. **7**), 190 fold selectivity APP over NOTCH) target the same binding site in the γ-secretase complex [21-24]. Finally, the non-sulfonamide NOTCH-sparing inhibitors targeting a putative nucleotide-binding domain Gleevec, ZM39923, sirtinol, (Fig. **8**) are also presumed to bind to the PSEN1 CTF based on evidence from competition experiments between ZM39923 and a photo-reactive ATP analogue [30-32]. It has further been proposed that non-transition-state GSIs may prevent substrate movement from the docking to the catalytic site, which would position their binding site in between the docking site and the active site [23]. In conclusion, while the exact binding pockets remain to be defined, all GSIs seem to interact with PSEN in the γ-secretase complex. 

### Substrate-Targeting γ-Secretase Modulators?

**Fig. (9):**
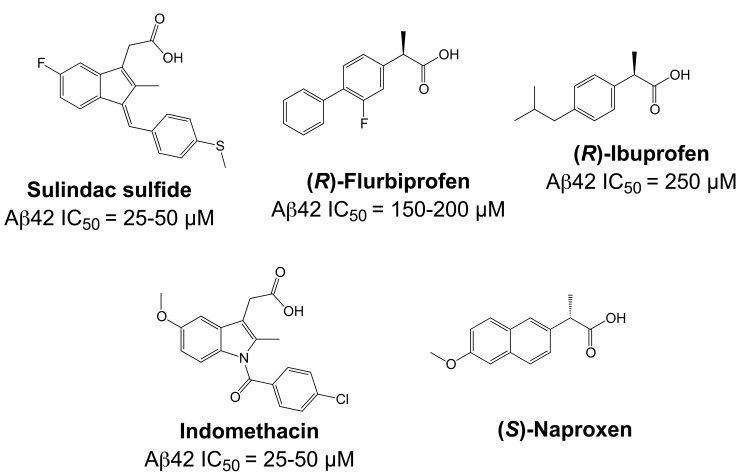
Structures of γ-secretase modulators in the class of NSAIDs. Only few NSAIDs are γ-secretase modulators, and naproxen represents an NSAID without this activity.

Substantial progress has been made to elucidate the molecular mechanism of GSMs. All GSMs that were initially discovered belonged to the drug class of non-steroidal anti-inflammatory drugs (NSAIDs) (Fig. **9**) [33, 91]. These compounds exert their principal analgesic effects through inhibition of cyclooxygenases (COX) [92]. However, it rapidly became clear that COX enzymes were not involved in the Aβ42-lowering activity of NSAID GSMs, and that COX-inhibition and Aβ42-lowering were distinct pharmacological entities [26]. Among other arguments, it was demonstrated that the NSAID GSM sulindac sulfide (Fig. **9**) could lower Aβ42 generation from COX-deficient fibroblasts [33].

Similarly, several non-COX targets of NSAIDs such as peroxisome-proliferator-activated receptors, lipoxygenases and nuclear factor κB [93], were shown to be unlikely involved in the selective modulation of Aβ42 levels by NSAID GSMs [94]. Of particular interest were findings by Zhou *et al.* that the small GTPase RhoA and its effector Rho-kinase (ROCK) were mediating the Aβ42-lowering activity of NSAID GSMs [95]. However, a subsequent study could not confirm key findings of this paper [96]. Overall, these studies argued against an indirect mechanism involving signal transduction to explain the GSM activity of NSAIDs (for a comprehensive discussion of the role of COX and non-COX targets in the Aβ42-lowering activity of NSAIDs see [26]).

In contrast, strong evidence has been provided that GSMs directly modulate γ-secretase activity by the demonstration that NSAID GSMs selectively inhibit Aβ42 and increase Aβ38 production in γ-secretase *in vitro* assays. Several groups have confirmed that both GSMs and inverse GSMs (compounds such as celecoxib that increase Aβ42 levels and decrease shorter Aβ species [97]) are active in these cell-free assays using either crude membrane preparations or partially purified γ-secretase complexes as a source of enzyme activity [91, 97-102]. Studies of GSMs in cell-free γ-secretase assays have further corroborated that GSMs do not affect ε-cleavage and generation of AICD and NICD domains over a certain range of concentrations [98, 100, 103]. Specifically, for the GSM sulindac sulfide it was shown that Aβ42 production was selectively and dose-dependently reduced at lower concentrations (20-100 µM) in a similar fashion to what had been observed in cell-based assays [98, 103]. However, at higher concentrations (above 600 µM), AICD generation was also impaired suggesting that in this concentration range sulindac sulfide acted similar to a non-selective GSI [98]. From these studies it was estimated that NSAID GSMs offer an approximately 10-fold concentration window where they inhibit Aβ42 generation without impairment of ε-cleavage and AICD formation [33, 98, 100, 103, 104]. For newer NSAID-type and non-acidic GSMs with nanomolar potencies such as E-2012 and GSM-1 (Fig. **14**, **16**), only few data concerning NOTCH selectivity in cell-based or *in vitro* assays have been published yet. A close analogue of GSM-1 (**21**, Fig. **16**) displayed an IC_50_ for Aβ42 inhibition of 640 nM (A(40 IC_50_ > 10 µM) without observable inhibition of Notch processing at the maximum assay concentration (10 µM) [105]. In addition, a potent non-acidic GSM (**9**, Fig. **14**) was shown not to affect proteolytic processing of NOTCH and a second γ-secretase substrate, E-cadherin, at concentrations 1000-fold above its Aβ42 IC_50_ (10 nM) [36].

**Fig. (10):**
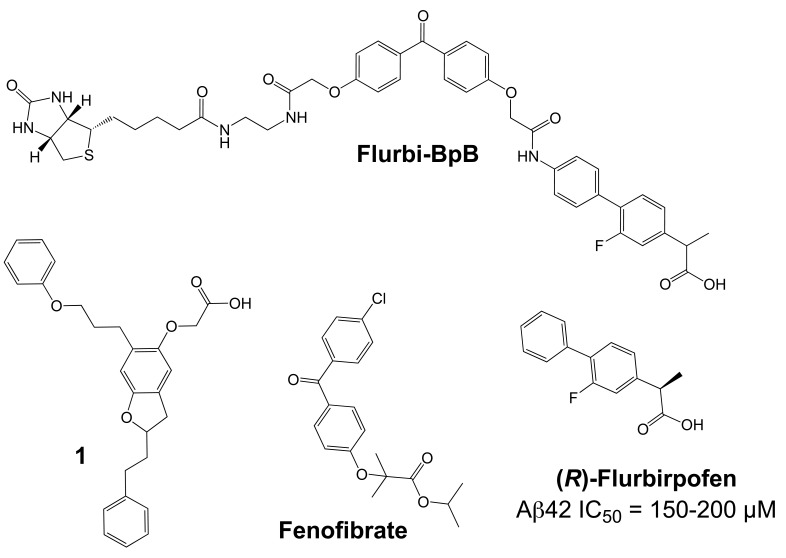
Substrate binders and the structure of Flurbi-BpB, a flurbiprofen-derived photo-reactive probe.

In addition to their activity in cell-free γ-secretase assays, several other observations supported the hypothesis that GSMs directly interact with the γ-secretase enzyme complex. First, studies using fluorescence lifetime imaging to monitor the spatial relationship between epitopes within PSEN1 provided evidence that GSM treatment might induce conformational changes within the γ-secretase complex [106, 107]. Second, certain PSEN1 and PSEN2 mutations associated with FAD have been demonstrated to strongly attenuate the cellular response to GSM treatment *in vitro* and in a transgenic mouse model of AD [35, 70, 108]. While these findings may have important consequences for future prevention studies in FAD patients [108], they further suggested that subtle structural and/or functional changes in the γ-secretase enzyme complex induced by the PSEN FAD mutations could affect the potency and efficacy of GSMs. Third, it has been shown that elongation of the N-terminus of the γ-secretase subunit PEN-2 by 10 amino acids increased Aβ42 production from the mutant γ-secretase complexes with a concomitant decrease in Aβ38 generation, a change in Aβ production that was reminiscent of the effect of inverse GSMs [109]. N-terminal elongation of PEN-2 was further associated with a decrease in the water accessibility of critical amino acids in PSEN1 that have been hypothesized to be exposed in the catalytic pore of the γ-secretase complex [53, 109]. Intriguingly, similar changes in the water accessibility of the catalytic pore were observed after treatment with the inverse GSM fenofibrate (Fig. **10**). This indicated that N-terminal extension of PEN-2 or treatment with inverse GSMs caused equivalent changes in the γ-secretase cleavage pattern through similar structural modifications of the catalytic center of γ-secretase [109]. Finally, in radioligand binding studies the NSAID GSMs sulindac sulfide and flurbiprofen were able to displace binding of both transition-state and benzodiazepine GSIs by non-competitive antagonism, providing further evidence for direct interaction of GSMs with the γ-secretase complex [22, 98].

In particular the radioligand competition experiments were highly suggestive of an allosteric binding site for GSMs within the PSEN proteins [26]. However, surprisingly, and in contrast to the findings with GSIs, the primary binding site of NSAID GSMs has been reported to reside in the substrate APP and not in one of the four subunits that form the γ-secretase enzyme complex. In a study by Kukar *et al.*, photo-activatable derivatives of GSMs were synthesized and employed for biochemical labeling studies [37]. These derivatives were based on the NSAID GSM flurbiprofen and the inverse GSM fenofibrate and incorporated benzophenone as a photo-active moiety and a biotin tag for biochemical purification (Flurbi-BpB, Fig. **10**). Given the low potency of the parent compounds, the photo-probes were present in the photo-affinity labeling assays in high concentrations (10-100 µM). Photolysis in the presence of partially purified γ-secretase activity demonstrated that none of the four γ-secretase subunits was labeled by the photo-probes. In contrast, a purified recombinant APP substrate consisting of the last 100 C-terminal amino acids of APP (APP-C100) was readily labeled by the photo-probes, and binding was competed by the parent compounds and other Aβ42-lowering NSAIDs such as sulindac sulfide and indomethacin, but not by NSAIDs lacking GSM activity such as naproxen (Fig. **9**). In addition, labeling of APP-CTFs and full-length APP was achieved in the presence of crude membrane preparations. An analogous recombinant C-terminal fragment of NOTCH-1 was also labeled by the photo-probes but with less efficiency requiring higher concentrations of the photo-probes. Mapping of the binding region of the photo-activatable GSMs within APP demonstrated binding to residues 29-36 of the Aβ domain. These residues form the beginning of the APP TMD with Gly29 being the first amino acid within the membrane, and they contain two consecutive GXXXG motifs, which have been implicated in APP dimerization and Aβ oligomerization (Fig. **3**) [75, 76, 110]. As described above, G/A mutations in the G_29_XXXG_33_ motif have been shown to selectively lower cellular production of Aβ42 peptides with a concomitant increase in Aβ38 production, a shift in the Aβ profile that is highly reminiscent of the effects induced by GSMs [75]. Finally, it was shown that other known Aβ-binding compounds such as the amyloid dye X-34 displayed characteristics of GSMs with selective Aβ42-lowering activity and a propensity to increase shorter Aβ species. From these observations it was concluded that potentially any small molecule binding to the Aβ domain of APP could function as a GSM [37]. Previously, a study by Espeseth *et al.* had provided support for this concept [111]. These authors presented benzofuran-containing compounds such as compound **1** (Fig. **10**) that interacted with full-length APP but not with an APP fragment lacking the Aβ domain in surface plasmon resonance binding assays. In γ-secretase *in vitro* assays using recombinant APP CTFs as substrate, some of the benzofurans including compound **1** behaved as GSMs with preferential inhibition of Aβ42 generation over Aβ40 generation. In addition, benzofurans were able to block γ-secretase cleavage of a substrate peptide that included residues 28-42 of the Aβ domain. Overall, these results suggested that benzofuran APP-ligands modulated γ-secretase activity in a GSM-like manner by binding to the Aβ domain of APP.

Taken together, these findings provided strong evidence that GSMs and inverse GSMs target the substrate APP instead of the γ-secretase enzyme [37]. However, of note, a recent NMR study questioned the specificity of the observed interaction between NSAID GSMs and APP [112]. In addition, several earlier observations do not easily conform to the concept of substrate targeting GSMs. First, enzyme kinetic analyses have demonstrated a mode of non-competitive inhibition of Aβ42 production for the NSAID GSMs sulindac sulfide and flurbiprofen in cell-free assays of γ-secretase activity [98, 100]. This indicates that the inhibitory effect of GSMs on Aβ42 production cannot be overcome by increasing substrate concentrations *in vitro*, which would be expected for compounds with a primary binding site within the substrate. Such non-competitive inhibition with respect to substrate has also been observed with GSIs from different structural classes including the prototypical transition-state inhibitor L-685,458 (Fig. **4**), which directly interacted with PSEN1 [85, 88]. Second, while GSMs do not impair γ-secretase cleavage close to the cytosolic membrane border and the release of intracellular signaling domains (ICDs) from several γ-secretase substrates in cell-based and *in vitro* assays [33, 98, 100, 103, 104], it has been convincingly demonstrated that GSMs do affect γ-secretase cleavage events in the middle of the NOTCH-1 TMD (S4-cleavage), which are analogous to the cleavage events in the APP TMD that generate the Aβ peptides (Fig. **3**). Okochi *et al.* [113] have identified Aβ-like peptides that are generated from NOTCH by γ-secretase with differing C-termini analogous to the Aβ peptides. Two peptides, Nβ21 and Nβ25, were predominantly produced from cells overexpressing NOTCH-1 in a ratio of 5:1 (Fig. **3**). Intriguingly, treatment with GSMs selectively lowered generation of the longer Nβ25 peptides without affecting levels of the shorter Nβ21 peptide [113]. Conversely, expression of PSEN1 mutants or treatment with inverse GSMs selectively increased Nβ25 levels. Importantly, treatment with 100 µM of the NSAID GSMs sulindac sulfide or indomethacin caused quantitatively similar reductions in the Aβ42 and Nb25 levels. This indicated that γ-secretase mediated cleavage events in the TMDs of APP and NOTCH-1 displayed similar sensitivity to GSMs [113]. The same group recently reported comparable results for another γ-secretase substrate, amyloid-precursor-like-protein 1 (APLP1) after treatment with inverse GSMs [114]. Third, signal peptide peptidase (SPP), an aspartyl I-CLiP homologous to PSEN, has been demonstrated to harbor a binding site for GSMs, and high concentrations (500 µM) of the NSAID GSMs sulindac sulfide and indomethacin (Fig. **9**) were able to shift the major cleavage site of a recombinant SPP substrate under cell-free assay conditions [115-117]. In contrast to γ-secretase, SPP functions as a homodimer without accessory proteins, and γ-secretase and SPP substrates do not show any overlap [118]. These findings seem to suggest that γ-secretase contains a binding site for GSMs that is conserved in SPP and, accordingly, should be present in PSEN. Finally, there is very little precedent in the literature for small molecule inhibitors targeting enzyme substrates [119]. This is most likely due to the difficulties in developing high-affinity compounds to small and linear epitopes with minimal 3D structure on the surface of proteins. Furthermore, the cellular concentration of a substrate is generally far in excess of its processing enzyme; this further complicates the development of potent compounds as higher drug concentrations are required to inhibit an enzymatic reaction by targeting the substrate. In fact, to the best of our knowledge, NSAID GSMs and the benzofuran APP-ligands (Fig. **9**, **10**) are the only small molecules that have been reported to distinctly target an enzyme substrate so far [37, 111]. Other reports of substrate-targeting molecules are restricted to macromolecules such as antibodies or smaller peptidic compounds [120]. One example is the humanized monoclonal antibody eculizumab, which binds the complement protein C5 with picomolar affinity and prevents its cleavage into pro-inflammatory products by C5 convertase activity [121, 122]. Nevertheless, the concept of GSMs as substrate targeting protease modulators could have far reaching implications for drug development beyond the potential use of GSMs in AD therapeutics. Very recently, two studies using other experimental approaches have also produced conflicting data. In the first study, binding of the NSAID GSM sulindac sulfide to recombinant Aβ42 was demonstrated by surface plasmon resonance and NMR spectroscopy providing support for targeting of the substrate APP [38]. In the second study, a close analog of the non-acidic GSM **9** (Fig. **14**) was coupled to a solid support (Affigel 10 agarose) and used for affinity chromatography. This affinity ligand retained predominantly PEN-2 and smaller amounts of the PSEN1 N-and C-terminal fragments [36]. However, the experimental design in the first study explored only the hypothesis that GSM might directly interact with the Aβ sequence, whereas in the second study it was not investigated whether the affinity ligand preserved its GSM activity after immobilization [36, 38]. Accordingly, additional studies with structurally diverse and smaller photo-affinity ligands based on GSMs with substantially improved potency are highly anticipated. Elucidation of the exact binding sites of GSM photo-probes could further be aided by tandem mass spectrometry [123].

Beyond that, the molecular details of how GSMs cause the observed shift in γ-secretase cleavage specificity from Aβ42 to Aβ38 remain largely unresolved. Potential explanations include GSM-induced changes in substrate presentation to the active site of γ-secretase. In this respect, defined movements of the APP TMD in the membrane might cause more frequent exposure of the γ38-cleavage site to the active center of γ-secretase and less frequent exposure of the γ42-cleavage site [26, 37]. Alternatively, and in accordance with the sequential cleavage model of Aβ generation, GSMs may strengthen the interaction between enzyme and substrate, and, consequently, enhance the turnover from Aβ42 to Aβ38 peptides [34, 62, 68]. Finally, expanding on the sequential cleavage model of Aβ generation, it has been proposed that GSMs interfere with the dimerization of the substrate APP and increase the accessibility of the γ38-cleavage site to γ-secretase. Such a mechanism could also favor the turnover of Aβ42 and the production of shorter Aβ species such as Aβ38 [38]. However, cell lines expressing APP variants with mutations in the G_29_XXXG_33_ dimerization motif, which lower Aβ42 production and reduce dimerization strength, were shown to be fully responsive to GSM treatment, arguing against the proposition that G_29_XXXG_33_ mutations and GSMs reduce Aβ42 production through the same molecular mechanism [124]. In any case, most of these explanations remain speculative at this point, and high resolution structural data of a GSM bound to the γ-secretase complex may be required to fully understand the molecular mechanism of GSMs.

## CHEMICAL DEVELOPMENT AND CLINICAL PERSPECTIVE OF γ-SECRETASE MODULATORS

**Fig. (11):**
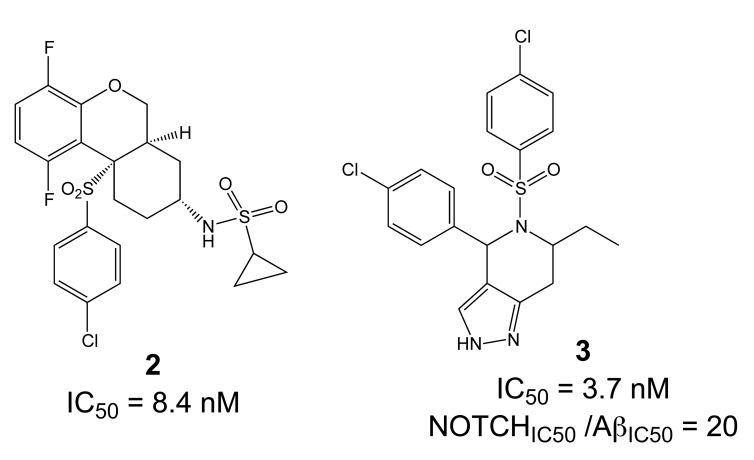
NOTCH-sparing sulfonamides.

The critical issue for clinical development of GSIs is impairment of NOTCH processing, potentially leading to severe side effects *in vivo*. None of the first generation peptidic GSIs elaborated after the Merck compound L-685,458 (Fig. **4**) [18] entered clinical trials, principally due to the poor pharmacokinetics and ADME properties inherent to peptides, which also applies to the docking site-targeting helical peptides [20]. Further development yielded second generation GSIs, which have entered clinical trials in the last years. Among the DAPT derivatives initiated by Elan/Lilly [125] with C-terminal modifications (Fig. **6**, LY-411,575, Compound-E), only one derivative, (LY-450,139, Semagacestat, Fig. **6**) has been evaluated in two phase III clinical trials in 2600 patients with mild to moderate AD. However, after a recent interim analysis of the trials, clinical development of LY-450,139 has been terminated. While the reasons for the failure of LY-450,139 are not known at this point, the suspension of the trials was attributable to enhanced cognitive decline and an increased risk of skin cancer in the treatment group as compared to placebo (Eli Lilly and Company, press release August 17, 2010). The third generation NOTCH-sparing GSIs with scaffolds typically centered on sulfonamides have progressed more rapidly into clinical testing, based on the assumption that drug dosage can be adjusted within a therapeutic window to avoid extensive NOTCH-related toxicity. The Bristol-Myers Squibb NOTCH-sparing compound BMS-708,163 (Fig. **7**) is currently in phase II (190-fold selectivity for APP over Notch) [126]. The 16-fold selective Begacestat (presently PF-5212362/ Pfizer, Fig. **7**) and a NOTCH-sparing GSI structurally similar to MRK-0752 (structure undisclosed, recent Merck GSIs are documented in [127] and Fig. **11**, compound** 2**) recently completed phase I [128-130]. Another NOTCH-sparing GSI from Elan, ELND-006, has entered Phase I (structure undisclosed, for further reading see reference [131] and compound **3** in Fig. **11**).

Preclinical studies indicate that gastrointestinal side-effects and effectiveness in lowering Aβ levels and amyloid plaque burden in AD mouse models are highly variable between the different classes of GSIs, with an overall better tolerability for NOTCH-sparing GSIs [24, 132, 133]. However, the distinction between early, non-selective GSIs and Notch-sparing GSIs remains somewhat ambiguous, and the degree of selectivity may to a large extent depend on the particular assays used to assess inhibition of APP and NOTCH processing. In fact, a recent re-evaluation of early, non-selective GSIs such as DAPT and compound-E concluded that they displayed significant selectivity for APP over NOTCH [134]. In addition, very little information is available concerning the selectivity of NOTCH-sparing GSIs towards other substrates of γ-secretase.

Importantly, the excellent NOTCH-selectivity of GSMs presents a clear alternative to GSIs, with outstanding perspectives for the development of safer disease-modifying drugs targeting the γ-secretase complex. The first GSMs were discovered in the class of NSAIDs (Fig. **9**), which, initially motivated by positive findings in epidemiological studies, have been investigated extensively in AD treatment and prevention studies [135]. Since the initial observation in 1990 that patients with rheumatoid arthritis under NSAIDs therapy were less likely to develop AD [136], numerous retrospective and prospective epidemiological studies over the past twenty years have supported the conclusion that chronic intake of NSAIDs is associated with a 25-80% lower risk of AD [137]. Several studies have further indicated that the duration of NSAID exposure is critical, with longer NSAID intake being associated with increased protection from AD [136, 138-142]. In addition, NSAID GSMs, in particular ibuprofen and indomethacin (Fig. **9**), have demonstrated therapeutic and preventive effects in several AD mouse models in chronic dosing studies [143-150] (for a detailed description of preclinical studies with NSAID GSMs in AD mouse models, please see [26, 135]). 

With Aβ42-lowering activity, and supported by positive epidemiological and animal studies, NSAID GSMs could be considered candidate drugs for treatment or prevention of AD. However, for a variety of reasons, the prospects for these drugs seem very limited today. (1) The mechanism of action of NSAID GSMs in AD remains unexplained. Besides COX, NSAIDs are known to engage a variety of molecular targets, and multiple anti-inflammatory and anti-amyloidogenic mechanisms have been proposed that could explain their preventive effects in humans and animal models (for a comprehensive discussion of these potential mechanisms see [34]). This severely complicates the interpretation of the epidemiological and preclinical studies with NSAIDs, and also raises the possibility that synergistic actions through several targets might be responsible for their efficacy in AD prevention. In the epidemiological studies, the NSAID GSMs ibuprofen and indomethacin (Fig. **9**) belonged to the most frequently prescribed NSAIDs. For example in a recent study by Vlad *et al.* in the US Veterans Health Care System, with almost 250.000 subjects by far the largest retrospective epidemiological study ever conducted, ibuprofen accounted for 20.9% of all NSAID prescriptions and was associated with a 40% risk reduction for AD in cases with ibuprofen use >5 years [141]. Accordingly, NSAID GSMs should have made a substantial contribution to the reported inverse correlation between NSAID use and AD risk as revealed in many epidemiological studies [138, 141]. Nevertheless, the published evidence does not support the conclusion that NSAIDs GSMs are more protective than NSAIDs without GSM activity. (2) Despite the generally mild toxicity profile of NSAIDs [151], the available data from clinical studies clearly indicate that these drugs are poorly tolerated in AD patients at standard prescription doses. Long-term inhibition of COX is associated with gastrointestinal and renal toxicity, and treatment studies with NSAIDs in AD have frequently suffered from high withdrawal rates [152, 153]. Moreover, a large, placebo-controlled, primary prevention trial for AD (ADAPT) with two non-GSM NSAIDs, naproxen and celecoxib, was suspended, and retrospective analysis showed signs of increased cardiovascular and cerebrovascular events in the NSAID-treated groups, indicating that serious risks associated with chronic NSAID consumption extend to elderly people without signs of clinical dementia [154]. (3) Finally, and most importantly, clinical studies conducted with the NSAID GSMs indomethacin and *(R)*-flurbiprofen (Figs. **9**, **10**) have not provided evidence for clinical efficacy in AD patients. While the two published trials with indomethacin remain ambiguous, with one trial reporting some slowing of symptom progression in AD patients and the second trial showing no significant differences in primary and secondary outcome measures, the results of these trials remain difficult to interpret because of their small size and high drop-out rate [152, 155]. In contrast, the recent failure of the large Phase III clinical trial with *(R)*-flurbiprofen (tarenflurbil) appears conclusive. *(R)*-flurbiprofen, the *(R)*-enantiomer of the racemic drug flurbiprofen, is only a weak GSM with an IC_50_ = 200 µM, but was considered a promising drug candidate for AD because of substantially reduced COX-activity and a reported benign safety profile. Interestingly, the carboxylic acid on aryl alkanoic acid GSMs (Fig. **9**) appears to be essential for the Aβ42-lowering activity, with ester analogues behaving frequently as inverse GSMs [97]. However, the early analysis of the structure-activity relationship (SAR) on aryl alkanoic acid COX inhibitors indicated that the analgesic activity is highly dependent on the absolute configuration of the carboxylic acid alpha-carbon, with the (*R*)-enantiomers being COX inactive. In a Phase II trial, *(R)*-flurbiprofen had shown some hints for clinical efficacy in a subgroup of patients with mild AD and high plasma drug levels [156]. This was followed by the largest Phase III trial ever conducted in patients with AD, in which 1684 patients with mild AD were treated with 800 mg *(R)*-flurbiprofen twice daily for 18 months. Unfortunately, this trial showed no effect of *(R)*-flurbiprofen on primary cognitive and functional outcome measures, and development of the drug was discontinued [157]. While the reasons for the failure of the trial may never be definitively known, one reasonable explanation is that *(R)*-flurbiprofen did not succeed because of insufficient brain penetration and target engagement. Limited blood-brain-barrier permeability for numerous NSAIDs is well documented, with typical brain to plasma ratios in the 1-5 % range, due to their inadequate physical properties related to molecular weight, total polar surface area and logP [91, 158]. In fact, a previously conducted phase I trial had not observed significant reductions in plasma and CSF Aβ42 levels of healthy volunteers treated with the highest dose of *(R)*-flurbiprofen used in the phase 3 trial [159]. Generally, free drug concentrations in the range of IC_50_ values are required to observe pharmacodynamic effects on a given target in brain. However, data from the Phase I trial and from previous animal studies indicated that maximal brain concentration of *(R)*-flurbiprofen were around 1-2 µM, around a 100-fold below the concentrations required to lower Aβ42 levels in tissue culture experiments. Thus, because of its poor pharmacological properties, *(R)*-flurbiprofen was likely not a credible drug candidate to test the hypothesis that GSMs are able to slow or modify the disease course in AD.

In conclusion, while NSAID GSMs have been important tools in basic and preclinical research, further exploration of NSAID GSMs for treatment or prevention of AD appears unlikely. For future clinical studies with GSMs, the lesson from the *(R)*-flurbiprofen trial seems to be that compounds with substantially improved potency and brain penetration are needed to efficiently modulate γ-secretase activity in brain. 

**Fig. (12):**
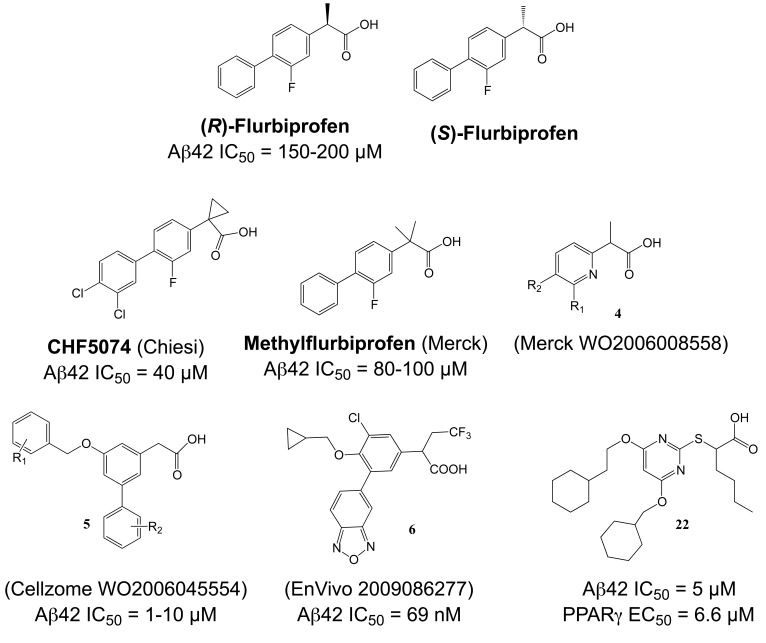
Ibuprofen-derived γ-secretase modulators.

While many NSAID-type and non-acidic GSMs with nanomolar to low micromolar potencies have been disclosed, only scant preclinical and no clinical data have been published for these second-generation GSMs. Chiesi has disclosed a variety of flurbiprofen analogues [160], including the GSM CHF5074 (Fig. **12**), with potencies often improved as compared to the parent compound flurbiprofen. The SAR analysis reported by Peretto *et al.* underlined the importance of the aromatic extensions and carboxylic acids, with modified esters turning GSMs into inverse GSMs [161]. The reported IC_50_ values for Aβ42 inhibition in the low to high micromolar range, although greatly improved with respect to (*R*)-flurbiprofen, nevertheless contrast with the nanomolar potencies typically obtained with current GSIs. For example, CHF5074 displayed an Aβ42 IC_50_ of 40 µM. Noteworthy the COX-activity has been nearly abolished due to the introduction of a cyclopropyl group in the carboxylic acid α-position (Fig. **12**) [161]. Chronic, 17-weeks treatment of the Tg2576 mouse model of AD with CHF5074 (375 ppm in the diet) starting at 9-10 months of age resulted in 50-75% reduction in amyloid plaque load in cerebral cortex and hippocampus, and a 50% reduction in SDS and formic acid extractable Aβ42 in brain [162]. The treatment was well tolerated, and no NOTCH-related side effects were reported. However, a previous short-term treatment study in the same mouse model at 5-7 months of age (100-300 mg/kg/day for 4 days by oral gavage) did not demonstrate changes in formic acid extractable Aβ42 levels in brain, raising some questions whether CHF5074 is centrally effective [163]. A second long-term treatment study with CHF5074 in a different APP-transgenic model demonstrated effects on amyloid plaque load and insoluble Aβ42 levels in brain, but also reported modest improvements in spatial memory deficits in the Morris water maze [164]. A first-in-man Phase 1a study with CHF5074 is currently ongoing (Bruno Imbimbo, personal communication). 

Merck reported on several geminal dimethyl NSAID analogues, with the best activities obtained with flurbiprofen derivatives such as methylflurbiprofen (Fig. **12**), which achieved 67% Aβ42 inhibition at 100 µM in HEK293 cells. The compound has been administered to the Tg2576 mouse model of AD for three days at 21 mg/kg/day. Only a 20% reduction in brain Aβ42 levels was observed although the compounds bioavailability and stability proved satisfactory. Further investigations indicated low brain penetration of the compounds, with an average brain to plasma ratio of 3% [165]. A subsequent patent [166] covered substituted pyridines (**4**, Fig. **12**) without disclosure of the obtained activities and pharmacokinetics. 

Cellzome reported interesting compounds with low micromolar activities obtained by further linking aromatics as phenyl ethers (**5**, Fig. **12**) [167]. Unfortunately, the pharmacokinetics and ADME properties for this scaffold are not available. However, EnVivo pharmaceuticals disclosed the activities of comparable aryl alkanoic acids from more than 3.000 phenyl ether analogues (**6**, Fig. **12**, with one example displaying an IC_50_ for Aβ42 inhibition of 69 nM in HEK293 cells [168]). Although the pharmacokinetics are not disclosed, the EnVivo lead compound EVP-0962 (undisclosed structure, Aβ42 IC_50 _= 114 nM) has been reported to reverse memory deficits in Tg2576 mice [169].

Other phenylether-based compounds have been reported as dual γ-secretase/PPARγ modulators [170]. Evidence indicates that PPARγ agonists might have multiple beneficial effects in AD both on pathological processes in the brain and on peripheral factors such as serum glucose levels and insulin sensitivity that constitute potential risk factors for AD [171]. The structure-activity relationship for the hybrid compound **22 (**Fig. **12**) has been investigated, pointing to a prevalent sensitivity of the scaffold in α-position to the carboxylic acid head group. In contrast, the aromatic backbone was fairly tolerant to modifications within the investigated substituents, although a significant preference for lipophilic moieties was observed [170].

**Fig. (13):**
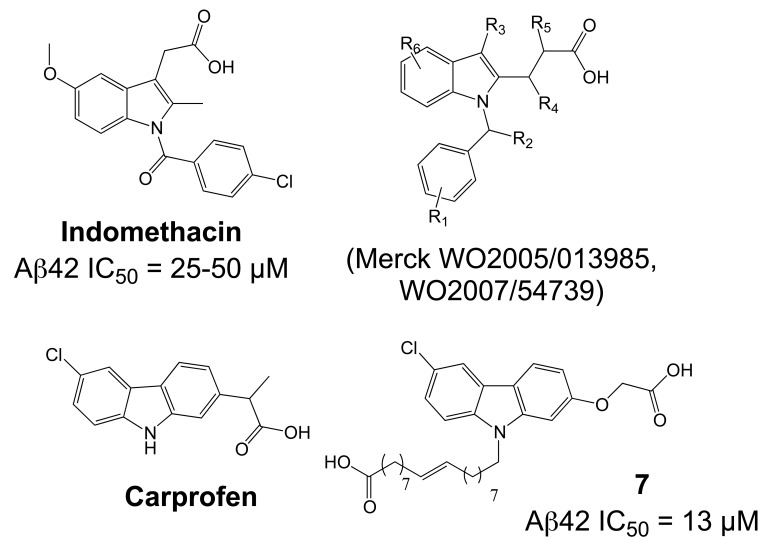
Indomethacin-derived γ-secretase modulators.

Along with the development of ibuprofen-derived GSMs, several groups reported indomethacin analogues (Fig. **13**). Merck disclosed several tetrahydroindole/indole-based structures in two patents without information about the potencies of the compounds [172, 173]. The carprofen-derived GSMs are better documented, with activities in the low micromolar range [174]. The authors reported a correlation between the compounds potency and lipophilicity, with the best activities obtained with N-alkylated derivatives (**7**, Fig. **13**), suggesting a potential membrane anchoring [175]. However, a specific interaction with the γ-secretase complex should not be ruled out, as exemplified by N-alkylated cannabimimetic indoles, whose alkylation correlated with increased binding affinity to the receptor [176].

**Fig. (14):**
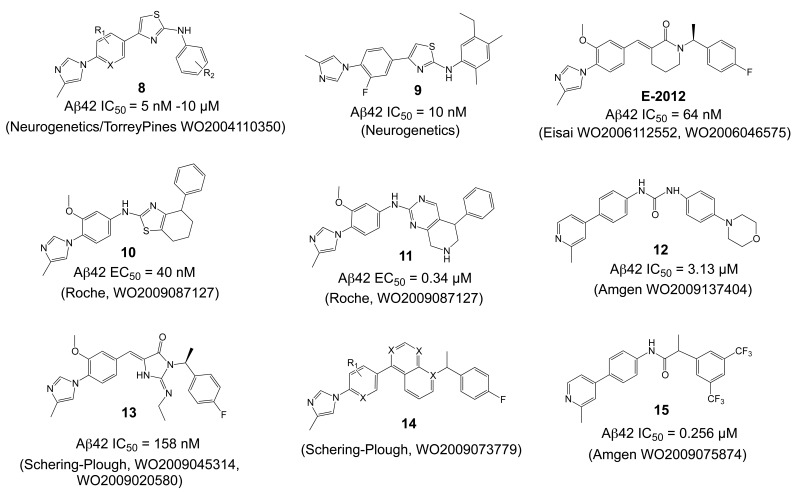
Non-NSAID γ-secretase modulators.

A novel class of compounds has appeared in the patent literature, with structures clearly distinct from the above-described NSAID-like GSMs, arising most probably from the screening of compound libraries. Neurogenetics (presently Neurogenetic Pharmaceuticals) disclosed the structures of the NGX modulators [36, 177], characterized by two aromatic moieties linked through an aminothiazole heterocycle (**8**, Fig. **14**). Importantly, the carboxylic acid functionality, which generally impedes the blood-brain-barrier crossing of NSAIDs, has been removed from these compounds. Most of the compounds disclosed in [177] displayed IC_50_ values in the 1-10 µM range with few compounds below 0.2 µM, and a later generation reaching low nanomolar potencies [36]. The scaffold appears unexpectedly tolerant to variations on the aromatic arms, but particularly sensitive to the N-alkylation of the 2-aminothiazole. A characteristic of these and other non-acidic GSMs is that they display less selectivity for Aβ42 and tend to lower Aβ40 in addition to Aβ42 levels, with a concomitant elevation of both Aβ38 and Aβ37 production [36, 178]. Most recently, pharmacokinetic and preclinical efficacy data has been reported for one compound from this series (**9**, Fig. **14**) [36]. After oral dosing of mice with **9** (50 mg/kg) an excellent brain/plasma drug concentration ratio of 0.93 was observed. Treatment of Tg2576 mice with the same dose for 3 consecutive days caused a significant 30% reduction in brain Aβ42 levels. Chronic long-term treatment of female Tg2576 mice from 8-15 months with **9** (50 mg/kg/day in food) reduced SDS-soluble as well as formic acid extractable Aβ40 and Aβ42 levels by ~ 50%, and amyloid plaque load in cortex and hippocampus by ~ 70%. No signs of NOTCH-related toxicity were apparent upon histological evaluation of the gastrointestinal tract [36]. Of note, the central thiazole heterocycle of this compound class might be subject to first pass metabolism [179]. For subsequent compounds developed in a research agreement with Eisai, the thiazoles were replaced by an α,β-unsaturated amide (Eisai E-2012, Fig. **14**) [180, 181]. The compounds display nanomolar activities for Aβ42 inhibition (E-2012 Aβ42 IC_50_ = 65 nM; [178]). However, neither the pharmacokinetics nor the selectivity towards NOTCH processing have been disclosed. Moreover, several α,β-unsaturated amides have been reported to be subject to conjugate addition leading to covalent adducts, which could substantially lower bioavailability of the compounds [182]. Nevertheless, Eisai resumed a phase I clinical trial with E-2012 after providing data showing that lenticular opacity, which was observed at high doses in rats, could not be detected in monkeys. 

Others have filed several patents with similarities in structures and activities to the Eisai/NGX compounds. The medicinal chemistry efforts were directed mostly at a better substance biocompatibility, and at modifications of the central α,β-unsaturated amide or thiazole linkers featured by the compounds described above. For instance, Roche disclosed the oxidation-resistant tetrahydrobenzothiazoles and pyrimidines (**10** and **11**, Fig. **14**) [183] with low to high nanomolar activities. Linkers based on iminohydantoins have been investigated at Schering-Plough and Merck, providing compounds with an Aβ42 IC_50_ of 158 nM, (**13**, Fig. **14**) [184-186]. They further disclosed compounds based on orthogonally-substituted fused aromatics (**14**, Fig. **14**), unfortunately without information on the obtained potencies and pharmacokinetics [187]. Amgen reported structures with a urea linker (**12**, Fig. **14**) that additionally replaced the terminal imidazole, typically observed in the Eisai and Roche compounds, by a pyridine (**12**, Fig. **14**) [188]. However, the potencies suffered from the change, leading to moderate micromolar IC_50_ values for Aβ42 inhibition. The pharmacokinetics for these compounds have not been reported. Nevertheless, the predictably unstable urea linker has been replaced by an amide in an Amgen patent [189], which also yielded increased potencies (**15**, Fig. **14**).

**Fig. (15):**
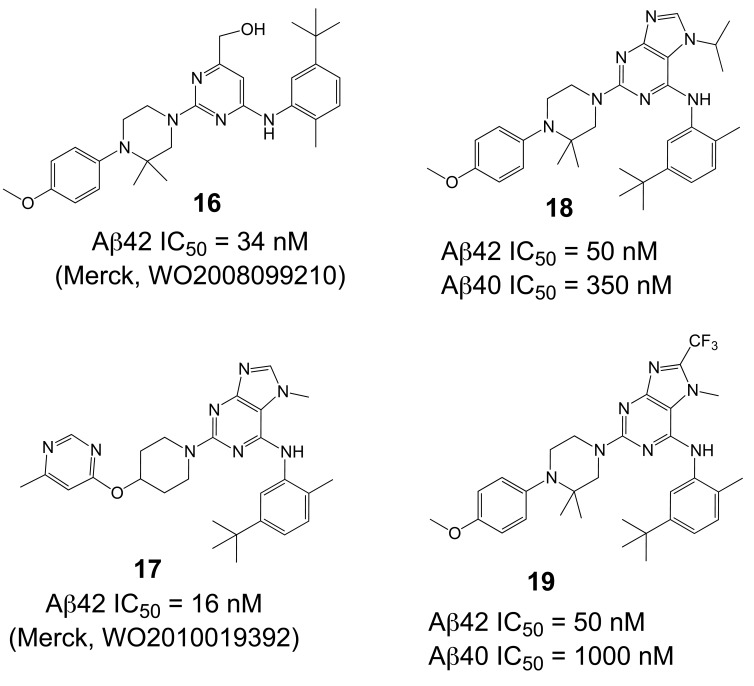
Structure of pyrimidine- and purine-based γ-secretase modulators.

Comparable structures have recently also been reported by Merck [190, 191], based on pyrimidine and purine heterocycles with low nanomolar potencies (**16** and **17**, Fig. **15**) [192, 193]. The SAR analysis of the pyrimidine structures indicated important hydrogen bonding contributions. The subsequent purines (**17**, Fig. **15**) displayed improved potencies, in addition to the successful removal of the metabolically unstable methoxylphenyl piperazine [190]. Acute oral dosing of an APP-transgenic mouse model of AD with 100 mg/kg resulted in a 70% reduction of Aβ42 levels in brain, and a satisfactory 34% brain/plasma concentration ratio. Interestingly, modifications of the GSM scaffold not only influenced the Aβ versus NOTCH selectivity, but also the Aβ42 versus Aβ40 selectivity, indicating a disconnect between the Aβ42 and Aβ40 inhibitory potencies (**18** versus **19**, Fig. **15**). However, the structures may show some overlap with known kinase inhibitors such as Gleevec (see Fig. **8**) [31]. However, data about potential off-target effects are not disclosed or not available. 

**Fig. (16):**
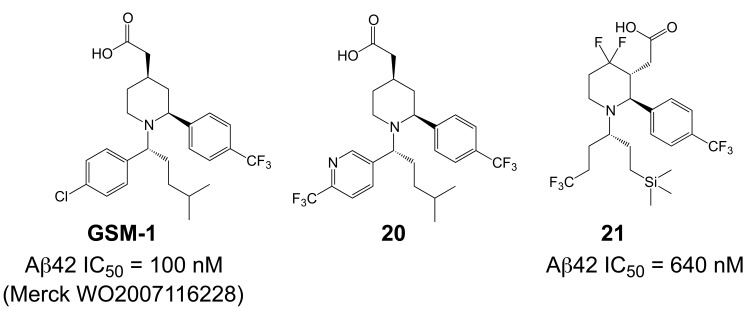
Piperidine-based γ-secretase modulators.

Up to now, the most convincing data demonstrating efficient modulation of γ-secretase activity in brain has been reported for another acidic modulator, GSM-1 (Aβ42 IC_50_ = 100 nM, Fig. **16**) [35, 194, 195]. A single dose of 3-30 mg/kg of GSM-1 was administered to APP-transgenic mice by oral gavage, and animals were sacrificed 4 h later. Soluble Aβ42 levels in brain showed a dose-dependent reduction and reached 50% at a dose of 10 mg/kg as compared to vehicle treated controls. Conversely, soluble Aβ38 levels increased in a dose-dependent manner, whereas Aβ40 levels showed no change [35]. These results indicate good bioavailability and brain penetration. Interesting pharmacokinetics have indeed been reported for fluorinated derivatives of GSM-1 (**21**, Fig. **16**) with adequate bioavailability and *in vivo* clearance. A satisfactory 53% brain/plasma drug concentration ratio has been described for compound **21** in rats, resulting in a dose-dependant lowering of Aβ42 brain levels with an 1 µM IC_50_ [105, 190]. The presented structures further suggest interplay between the lipophilicity and steric bulk of the substituents. An independent group has reported additional SAR on compounds derived from these Merck GSM-1 piperidine compounds, showing an excellent 74% brain/plasma concentration ratio for compound **20** (Fig. **16**) [196]. Whether any of these potent GSMs with improved biocompatibility have entered early phases of clinical testing is unknown at this time.

## CONCLUSIONS

Multiple lines of evidence indicate that oligomerization and accumulation of toxic Aβ42 peptides in brain is a triggering event in Alzheimer's disease. The aspartyl protease γ-secretase catalyses the last step in the cellular generation of Aβ42 peptides and is a high-priority therapeutic target in AD. γ-Secretase modulators (GSMs) are small molecules that selectively lower Aβ42 levels and shift the production of Aβ peptides to shorter and less toxic species. In contrast to non-selective γ-secretase inhibitors, GSMs do not affect proteolytic processing of other physiologically important substrates of the γ-secretase complex including the NOTCH receptor. Consequently, GSMs might be candidate drugs for future prevention trials in AD that aim to halt the pathological process before cognitive impairment occurs. The first GSMs were discovered in the class of non-steroidal anti-inflammatory drugs (NSAIDs). However, NSAIDs with GSM activity suffer from low potency and brain permeability, and toxicity associated with cyclooxygenase inhibition. Hence, while NSAID GSMs have been important tools in basic and preclinical research, further exploration of NSAID GSMs for treatment or prevention of AD appears unlikely. Most importantly, the lesson from the failed phase III clinical trial with *(R)*-flurbiprofen in AD seems to be that compounds with substantially improved potency and brain penetration are needed to efficiently modulate γ-secretase activity in brain. A major obstacle for the design of more potent GSMs with improved biocompatibility has been the lack of a three dimensional structure of the γ-secretase complex, impeding rational drug design. Nevertheless, recently, GSMs with nanomolar potency and good brain exposure have been described. These compounds can be divided in two major classes, acidic GSMs that retain some structural similarities to NSAIDs and non-acidic GSMs based on bridged aromatics. Important questions remain concerning these second-generation GSMs including their exact molecular mechanism of action and their efficacy in preclinical mouse models of AD, particularly with respect to behavioral endpoints. Given the rapid progress in GSM development documented in recent publications and patent applications, early-phase clinical trials of potent GSMs are to be expected soon.

## Figures and Tables

**Table 1.** Non-Exhaustive list of γ-Secretase Inhibitors and Modulators with Representative Classes and Scaffolds

γ-Secretase Inhibitors					γ-Secretase Modulators	
Docking Site^a^	Active Site^a^	Allosteric Site^a^		Nucleotide-Binding Site^a^	Allosteric Site^a^	Substrate Binding^a^
D-10 D-13	L-685,458 III-31-C	DAPT Cpd-E LY-411,575 LY-450,139 BMS-433,796	MRK-560 GSI-953 BMS-299,897 BMS-708,163	Gleevec Sirtinol ZM-39923	Sulindac sulfide Indomethacin CHF5074 EVP-0962 E-2012 GSM-1	Flurbiprofen-BpB
Val-Gly-*Aib*-Val-Val-Ile-*Aib*-Thr-Val-*Aib*-X	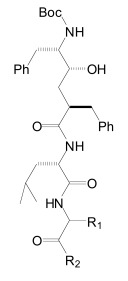	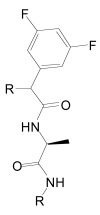	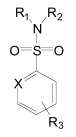	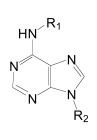	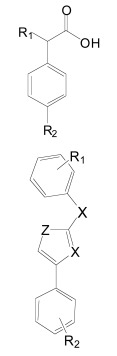	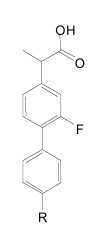

a Putative Binding Site of Compounds.

## ABBREVIATIONS

Aβ: = Amyloid-β peptide
AD: = Alzheimer’s disease
ADAPT: = Alzheimer’s disease anti-inflammatory prevention trial
ADME: = Absorption Distribution Metabolism Elimination
AICD: = APP intracellular domain
APH-1: = Anterior pharynx defective-1
APLP1: = Amyloid-precursor-like-protein 1
APP: = Amyloid precursor protein
ATP: = Adenosinetriphosphate
BACE1: = β-site APP-cleaving enzyme 1
C99: = C-terminal 99 amino acid fragment of APP
CHAPSO: = 3-[(3-Cholamidopropyl) dimethylammonio]-2-hydroxy-1-propanesulfonate
CTF: = C-terminal fragment
COX: = Cyclooxygenase
DAPT: = N-(N-(3,5-difluorophenacetyl)-L-alanyl)-S-phenylglycine-t-butylester
DSL: = Delta/Serrate/LAG-2 (family of proteins)
FAD: = early-onset familial Alzheimer’s disease
Flurbi-BpB: = Flurbiprofen with a photoactive benzophenone moiety and a biotin tag
GSI: = γ-Secretase inhibitor
GSM: = γ-Secretase modulator
HEK: = Human embryonic kidney
HIV: = Human immundeficiency virus
HTS: = High Throughput Screening
ICD: = Intracellular cytoplasmic domain
I-CLiP: = Intramembrane-cleaving protease
µM: = Micromolar
Nβ: = Aβ-like NOTCH peptide
NEXT: = NOTCH extracellular truncation
NICD: = Notch intracellular domain
NFT: = Neurofibrillary tangles
NSAID: = Non-steroidal anti-inflammatory drug
NTF: = N-terminal fragment
PEN-2: = Presenilin enhancer-2
PSEN1/2: = Presenilin1/2
ROCK: = Rho kinase
SAR: = Structure-activity relationship
SDS: = Sodium dodecyl sulphate
SPP: = Signal peptide peptidase
TMD: = Transmembrane domain
